# The Potential for Using Extended Reality Technology in Interdisciplinary Case Discussions and Case Planning in Stereotactic Radiosurgery: Proof-of-Concept Usability Study

**DOI:** 10.2196/36960

**Published:** 2022-06-01

**Authors:** Swathi Chidambaram, Maria Chiara Palumbo, Vito Stifano, John McKenna, Alberto Redaelli, Alessandro Olivi, Michael Apuzzo, Susan Pannullo

**Affiliations:** 1 Department of Neurosurgery Weill Cornell Medical College New York, NY United States; 2 Department of Electronics, Information and Bioengineering Politecnico di Milano Milan Italy; 3 Fondazione Policlinico Universitario Agostino Gemelli Department of Neurosurgery Catholic University Rome Italy; 4 Department of Radiation Oncology New York Presbyterian Hospital New York, NY United States

**Keywords:** mixed reality, augmented reality, extended reality, HoloLens, interdisciplinary teams, virtual reality, brain tumour, tumor, radiosurgery, surgery

## Abstract

**Background:**

Extended reality (XR) is a term that captures a variety of techniques, such as augmented reality (AR) and mixed reality (MR), which allow users to interact with virtual models in real time. This technology has an emerging role in several applications within neurosurgery. XR can be useful in enhancing how radiosurgical cases are planned. Multidisciplinary team (MDT) review is an essential part of the radiosurgery case planning process; during case discussions, patient images are reviewed, usually in 2D or 3D modifications. The current commercially available platforms for this review need improvement.

**Objective:**

We describe a novel visualization application, titled “NeuroVis” by our development team, which uses an XR Microsoft HoloLens headset to provide an interactive 3D visualization of a patient’s neuroanatomy in stereotactic surgery (SRS) case planning discussions.

**Methods:**

We present examples of 6 common radiosurgery indications to demonstrate the utility of NeuroVis to solve common visualization hurdles in MDTs.

**Results:**

The utility of NeuroVis is demonstrated through 6 common brain tumor SRS cases as a proof-of-concept illustration of the utility of NeuroVis to enhance radiosurgery case discussion by improving visualization of the standard neuroimaging used in radiosurgery treatment planning by MDTs.

**Conclusions:**

The NeuroVis application provides several interactive features that produce an enhanced ability to place participating members of an interdisciplinary treatment team on the same visualization plane. This technology, by facilitating team discussions and case review, has the potential to improve the efficiency, efficacy, and safety of radiosurgery treatment planning and, as a result, to optimize patient care.

## Introduction

Neurosurgery involves complex anatomy, high levels of accuracy, and extreme precision. Image-guided neuronavigational technologies are often used in neurosurgical procedures; these platforms have undergone several developments in recent decades. Extended reality (XR) technologies, such as augmented reality (AR) and mixed reality (MR), which allow the viewer to merge a virtual environment into a real, physical environment, have emerging roles in the future of neurosurgery [1-3]. XR allows for visualization and virtual manipulation of anatomical structures beneath the surface anatomy, thereby aiding in surgical planning and education [4-7]. Furthermore, XR technologies allow surgeons to view 3D holographic reconstructions of anatomical regions of interest, thus improving upon the simple 2D views that are offered by most current neuronavigation systems. XR approaches, including AR and virtual reality, have already been studied in the neurosurgical subspecialties of spine, tumor, vascular, and pediatrics [8-12]. Importantly, however, this technology has not yet been applied to the field of brain stereotactic radiosurgery (SRS).

SRS is a highly interdisciplinary subspecialty in neurosurgery where brain imaging is crucial in treatment planning and delivery. Cases are often reviewed in multidisciplinary team (MDT) conferences, where cases are presented and imaging is reviewed. This MDT approach is an essential part of radiosurgery treatment planning, and its features in brain SRS have been previously described [13]. Relationship of radiosurgery targets among each other and with “critical structures” is key in creating safe treatment plans and in optimizing the efficiency of treatment delivery. These relationships are best appreciated in a 3D space. A particular challenge of MDT discussions in radiosurgery conferences is that communication is limited by participants’ varying ability to mentally convert 2D radiological images into 3D anatomical views. “Simulated” 3D projections are often presented as a surrogate for real 3D views in an effort to depict anatomy via rotation of images to mimic 3D space. To address this issue, we created an application called NeuroVis, which can provide an accurate and interactive 3D visualization of a patient’s neuroanatomy that can be displayed during SRS case planning discussions through the use of an XR headset. To our knowledge, XR technology has not yet been integrated into the MDT case discussions. In this proof-of-concept technical note, we describe and demonstrate, through selected figures and a video (Multimedia Appendix 1), how NeuroVis could enhance radiosurgery case planning discussions among MDTs for 6 common brain tumor case scenarios.

## Methods

### Overview

All patient radiographic images were anonymized prior to their use. A commercially available XR headset, Microsoft HoloLens, was used for the visualization and interaction with virtual holograms (Figure 1). The HoloLens is a head-mounted display with video-transparent lenses and has an untethered and wireless design.

**Figure 1:**
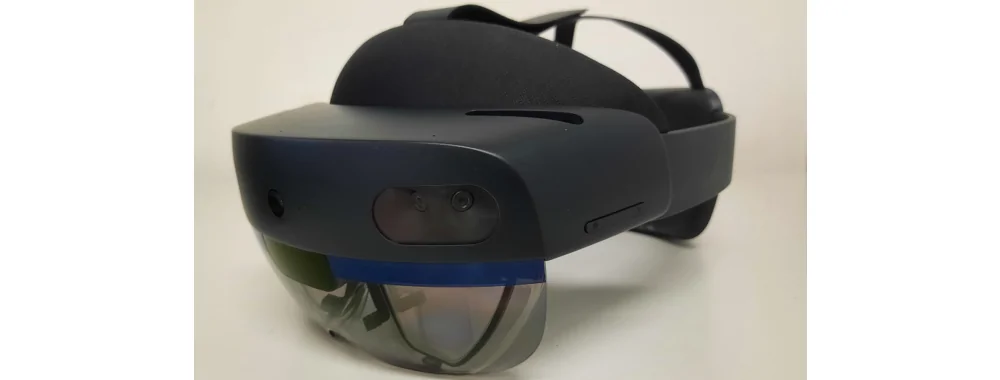
Microsoft HoloLens headset.

### Patient-Specific Hologram Creation

The NeuroVis application was designed with Unity 3D (version 2019.2.17; Unity Technologies), a game engine software, and used in conjunction with the Mixed Reality Toolkit (MRTK), a Microsoft-driven library that provides a set of components and features used to accelerate cross-platform MR application development in Unity 3D.

Patient-specific 3D models of the brain were created from both anonymized magnetic resonance imaging (MRI) and computed tomography (CT) acquisitions. The segmentation of the different brain structures was accomplished using software developed by Brainlab, integrated in the planning procedure, and 3D Slicer, an open-source software platform for medical image processing, applying different segmentation tools. The exported 3D models were imported into the virtual scene and supplemented with scripts to allow the holograms to be interactable with the user’s hands (move, scale, and rotate). Moreover, several tools and interactive features were developed to allow the user to (1) hide the different anatomical structures independently, (2) isolate lesions and planning treatment volumes (PTVs), (3) visualize axial, coronal, and sagittal MRI or CT planes overlaid on the 3D model, (4) manipulate handheld clipping planes that allow one to visualize cross-sections of the model in real time, and (5) change the opacity of each anatomical structures independently within the model. The holographic interface was designed closely with the end user in order to be user-friendly, effective, and useful during the procedural discussion. The application proved to be working for both HoloLens (versions 1 and 2; Microsoft Inc), making minor changes to the MRTK profile settings before the application is built and deployed on the device.

Regarding the visualization of raw MRI or CT images, a volume rendering open-source code implemented for Unity was adapted to work in the HoloLens and specifically adjusted for the application. A 2D projection of a 3D discretely sampled data set (MRI or CT volume) was displayed on 3 orthogonal planes to reproduce the sagittal, coronal, and axial view. All the anatomical planes can be scrolled throughout the imaging volume using hand gestures on virtual sliders. The method used to render 3D data is the raymarching technique. The way in which all the sampled volume values were combined and then displayed on the output-rendered image was determined using a direct volume rendering with a 1D transfer function. As the displayed images are a projection of a 3D volume on a 2D plane and not a preacquired stack of 2D images, the user is able to manipulate and choose every possible spatial orientation of a plane encompassing the imaging volume.

In total, 6 clinical scenarios are presented, demonstrating the use of NeuroVis in SRS case discussions.

### Ethical Considerations

The Office of Research Integrity at Weill Cornell Medicine conducted a review of this project and determined that it did not constitute human subjects research and therefore did not require further institutional review board approval or exemption, as identifiable private information was not being obtained or used.

## Results

### Overview

The presented cases represent common applications for radiosurgery; we chose a variety of neuro-oncologic scenarios, as these represent the most usual indications in our practice, and ones in which the value of NeuroVis was most apparent to us in developing this tool. The associated multidisciplinary case discussion process is described for each situation, to allow NeuroVis use to be understood in context.

### Case Scenario 1: Postoperative Resection Cavity

Patients are commonly referred for radiosurgery following resection of a brain metastasis, with the goal of minimizing the risk of local recurrence. Postoperative SRS addresses the surgical cavity, minimizing the risks of wider field irradiation [14,15]. The radiosurgical target can be large and in proximity to eloquent structures and organs at risk (OARs) [16]. In our multidisciplinary radiosurgery conferences, the neurosurgeon and radiation oncologist, along with the dosimetry or physics team, develop a plan for postoperative SRS based on pre- and postoperative imaging. The formulation of the treatment plan is typically based on discussions of the MRI and CT scans on a 2D screen (Figures 2A and 2B). Introducing NeuroVis in this step allows each viewer to see and interact with the same 3D hologram when discussing cases, and improves the understanding of the relationships between key structures and the resection cavity. For instance, the hologram viewed and manipulated through the XR device gives a better impression of the size and shape of the resection cavity and allows the neurosurgeon to explain operative approaches to the team. As seen in Figure 2C, the XR technology also allows for better visual approximation of the resection cavity and nearby OARs. Furthermore, the operative corridor can be more clearly imagined when the cerebrum is faded away. NeuroVis can optimize the visualization of volumes in the postoperative setting (Figure 2D). With NeuroVis, the MRI planes can also be integrated with the model to further understand the 2D to 3D transition (Figure 2E). A feature called the clipping plane allows for better understanding of the surface anatomy in relation to the treatment region (Figure 2F). All of these unique features of using NeuroVis in the SRS case discussion setting can help improve our ability to visualize and plan these common SRS cases.

**Figure 2:**
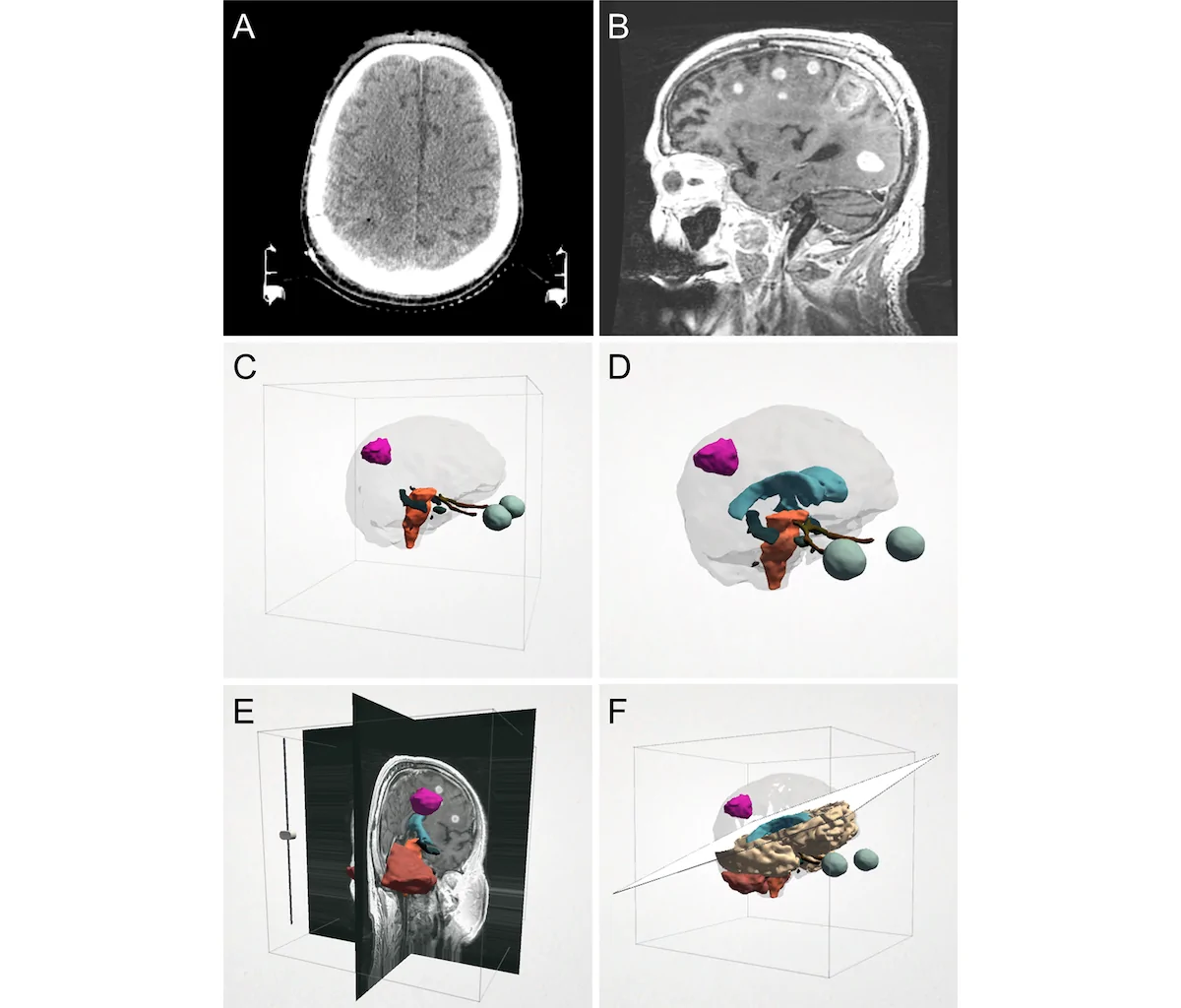
NeuroVis holograms for a patient undergoing stereotactic radiosurgery planning for treatment of a postoperative resection cavity.

### Case Scenario 2: Resection Cavity With Additional Metastasis

In cases in which a patient with multiple brain metastases undergoes resection of 1 or 2 dominant lesions, the patient often undergoes postoperative radiosurgery for the resection cavities and the remaining unresected lesions. In these situations, the maximum dose constraints to OARs are an important consideration given the multiple targets, of which one or more can be large, and the potential for overlapping treatment arcs [17,18]. Using NeuroVis, OARs and radiosurgical targets can be isolated and combined in various ways to help maximize the safety of treatment plans by helping the dosimetrists and physicists see these key relationships in a dynamic way (Figure 3A). Furthermore, incorporating MRI planes with holograms during planning can be helpful in understanding the relationship between the resection cavity and lesions (Figure 3B). This new way of seeing and planning can help determine whether lesions can be clustered for staged treatments as each lesion and resection cavity can be virtually isolated to form groupings [17]. The clipping plane feature allows us to view the relationship with the scalp and helps us create plans that minimize the scalp dose with grouping of lesions and fractionation (Figure 3C) [19].

**Figure 3:**
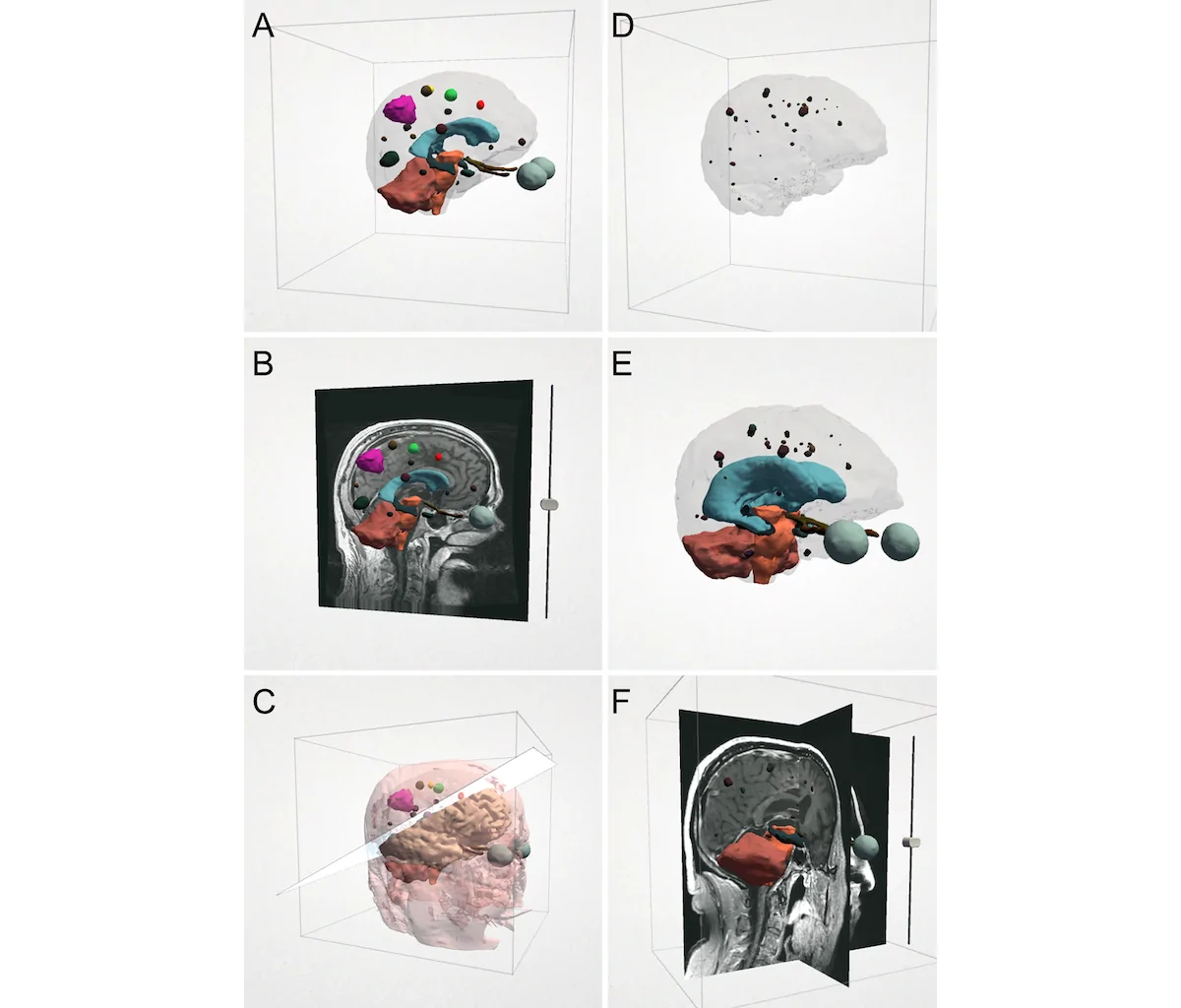
NeuroVis holograms for a patient undergoing stereotactic radiosurgery planning for treatment of a resection cavity with metastasis (A-C) and for a patient with multiple intracranial metastases (D-F).

### Case Scenario 3: Multiple Brain Metastases

Patients with multiple intracranial metastases, previously treated with whole brain irradiation, now commonly undergo SRS [20-23]. Furthermore, radiosurgical treatment for many brain metastases is now feasible [22,24,25]. The main visual challenge in planning cases with several metastases is understanding the topography or spread of the many lesions intracranially. NeuroVis can enhance these discussions by allowing all practitioners on the team to view the patient’s tumor burden and topography in 3D holograms (Figure 3D). Visualizing and reviewing cases with NeuroVis provide an appreciation of the clusters of lesions that might be present, especially as some lesions are too small to easily understand purely on 2D MRI evaluation. Moreover, the hologram in a XR headset allows for an interactive view of multiple lesions where MRI planes can be combined to show the transformation from 2D to 3D visualization (Figure 3F). The features in NeuroVis make it possible to more accurately understand the proximity of lesions to OARs (Figure 3E). Furthermore, changing the opacity of certain anatomical structures such as the ventricles and brain stem can be helpful in elucidating their relationships to adjacent subcentimeter lesions.

### Case Scenario 4: Brain Stem Metastases

A particularly challenging SRS case scenario is that of a patient with single or multiple brain stem metastases. The previously prevalent pessimism regarding the outcomes of patients with brain stem metastases, which resulted in the use of whole brain radiation rather than stereotactic radiosurgery in these patients, has been challenged by the favorable results shown in several single and multi-institutional case series of patients treated with SRS for brain stem metastases [26-29]. Nevertheless, these remain very challenging cases to treat with SRS given the strict dose constraints associated with the brain stem, a key OAR. In planning discussions for brain stem metastases, the 3D orientation, shapes, and clustering of small brain stem lesions are often poorly visualized with traditional imaging modalities on a 2D screen. With NeuroVis, the opacity of the brain stem itself can be altered to allow for a clearer understanding of the relationship of the lesions within the brain stem anatomy (Figure 4A). Similar to the technique used in other clinical cases, the axial, coronal, and sagittal MRI planes can be overlaid with the hologram to better visualize the relationship between the 3D oriented model and 2D MRI contours (Figure 4B). Here too, the clipping plane feature allows for delineation of the relationship between the contour of the brain stem itself and the lesions within (Figure 4C). In addition, by fading the cerebrum, we can gain a greater appreciation of the depth of the lesions and their relationship to the surface anatomy. These new techniques of manipulating and visualizing these challenging lesions can be used to optimize dosimetry and safety of the treatment plan.

**Figure 4:**
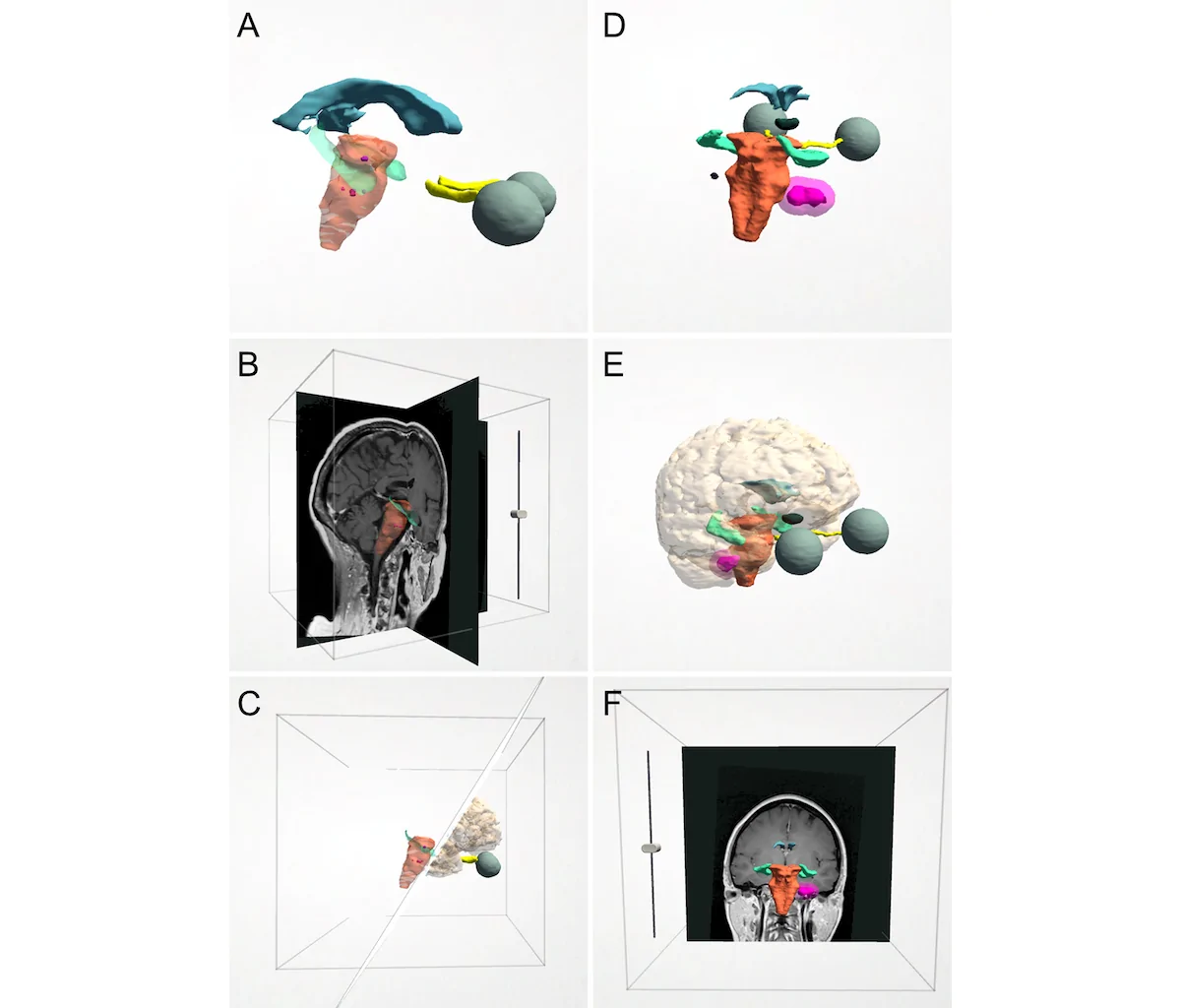
NeuroVis holograms for a patient undergoing stereotactic radiosurgery planning for treatment of a brain stem metastases (A-C) and for a patient with a vestibular schwannoma (D-F).

### Case Scenario 5: Vestibular Schwannoma

Vestibular schwannomas (VSs) are often treated with radiosurgery owing to excellent rates of tumor control and safety. Hearing preservation is a key goal; studies suggest a better chance of hearing preservation with radiosurgery than with observation for VS [30]. Limiting the radiation dose to the cochlea during SRS has been shown to be important in hearing preservation [31,32]. NeuroVis shows the VS contours in detail, allowing for a better understanding of the tumor’s proximity to the cochlea to facilitate planning. Isolating a holographic depiction of the lesion and PTV allow us to see its proximity to the brain stem and cochlea, the closest OARs (Figure 4D). Similar to the case of the multiple brain stem metastases, the deep-seated location of a VS can be better understood by changing the opacity of the overlying cerebrum to further elucidate the relationship between this lesion and its surrounding structures (Figure 4E). Adding the MRI planes also augments the ability to identify potential surgical corridors (Figure 4F) if surgery is still under consideration for a patient as a potential treatment option.

### Case Scenario 6: Intraventricular Lesion

In planning radiosurgery for intraventricular lesions, we acknowledge that the lateral ventricles are complex semicircular structures that are often poorly understood on a 2D radiograph. A 3D holographic representation of the ventricles is thus inherently useful in understanding their anatomy (Figure 5A). When combined with various MRI planes, this visualization of the intraventricular pathology can be further enhanced with NeuroVis. Fading the opacity of the ventricles allows for visualization of the target and its voluminal occupancy of the ventricles in 3D space (Figure 5B). Of note, this case demonstrates some new challenges that arise in terms of segmenting the MRI scans to create a 3D model. Heterogenous enhancement of this intraventricular meningioma complicated the 3D segmentation of the ventricle, which was needed to create an accurate hologram (Figure 5C)—a hurdle that was overcome with use of manual segmentation using tools available on 3D slicer.

**Figure 5:**
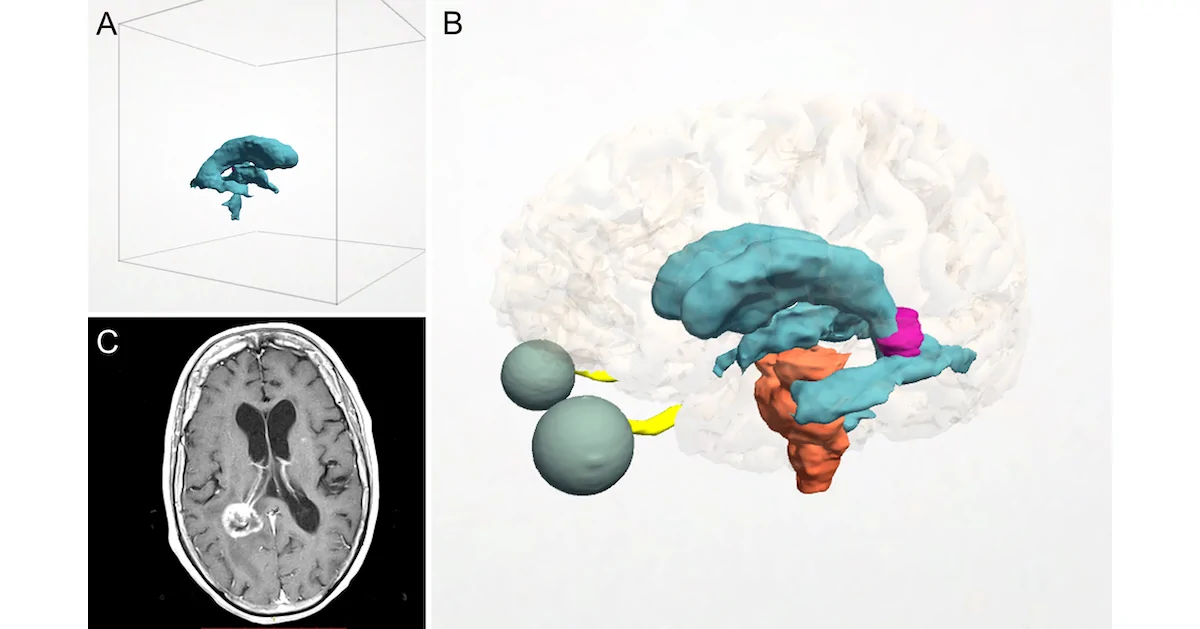
NeuroVis holograms for a patient undergoing stereotactic radiosurgery planning for treatment of an intraventricular lesion.

## Discussion

### Principal Findings

In this proof-of-concept technical note, we have demonstrated that NeuroVis can present imaging data in a better way by placing all users in an MDT on the same visualization plane when discussing cases. It also helps us understand the size, shape, and distributions of lesions more clearly and in 3D. This technology makes the relationship between target lesions, OARs, and surface anatomy appear more obvious. While there are challenges in scaling the use of this technology, the potential benefits entice us to continue to work toward this goal. There are also several areas of potential future expansion in this field through the integration of machine learning and in improvements in automatization of brain segmentation. In addition to facilitating technical discussions, NeuroVis may help optimize SRS case planning by creating more efficient treatment planning workflows, and ultimately optimize radiosurgical treatment delivery efficiency and safety, with an end goal of improving patient outcomes and quality of care.

### Challenges and Limitations

As with all new technologies, there are challenges to scaling the implementation of XR use in radiosurgical case planning. For instance, as previously mentioned, accurate 3D segmentation is a critical step in creating precisely reconstructed holograms to be integrated into the HoloLens headset from 2D MRI scans. Software such as FreeSurfer, Vbm, Ibaspm, and others provide the ability to segment the normal brain [33,34]. However, performing segmentation of the brain for patients with brain tumors is more complicated and often requires the combination of multiple modalities [34,35]. Most radiosurgical treatments rely on effective detection and precise segmentation of lesions. Thus, in radiosurgery, there are opportunities for further innovation with many methods of automatic brain segmentation based on deep learning technologies. These methods are being developed for pretreatment segmentation of gliomas and brain metastases for the purpose of maximizing safety during high-dose radiation treatments [35]. These advanced methods of automatic segmentation can also be applied to address the segmentation challenges that sometimes arise when creating holograms for XR use in SRS.

Other challenges to consider relate to the headset. These include the hurdle of acquiring the somewhat rare headset devices on a large scale at academic centers. The authors are optimistic that with the advent of newer, cheaper, and scalable production of XR headsets, this issue will be easily addressed in the near future. Regarding the portability and comfort of wearing such devices, an improvement in terms of design and fit has already been obtained using HoloLens versions 1 and 2 (Microsoft Inc). Despite a small improvement in the quality of the rendered holograms, the comfort of the second version of the device makes this technology much more suitable in case discussion where the device needs to be worn for many minutes. However, given the expanding market for XR in both medical and nonmedical sectors, design evolution is expected to occur rapidly.

In order for this technology to be successfully incorporated in case discussions, each institution must establish a methodology of transferring the requisite imaging data for each patient among team members and allocating tasks in creating and uploading holograms prior to MDT case conferences. In our preliminary experience with creating and executing these holograms, the workflow can be successfully established with a few team meetings to delegate tasks.

### Future Directions

Many of the challenges in scaling the use of this technology in fact highlight opportunities for growth and expansion. Having demonstrated proof of concept for NeuroVis in this technical report, in future studies, we plan to compare NeuroVis to conventional 3D imaging modalities such as 3D MRI angiography or 3D CT angiography, which are frequently used in cases that involve complicated neuroanatomical correlates to further investigate and measure the added benefit of our application in interdisciplinary case discussions and planning of SRS cases. Furthermore, a future study with questionaries and usability scores would be a meaningful next step to further test this application. Moreover, there is emerging literature on the changes in neurosurgical practice brought on by the onset of the COVID-19 pandemic [36]. The most prevalent changes as a result of this pandemic to health care and neurosurgery hinge on the increased use of telemedicine and remote tele-immersive conferencing [37]. This shift to remote communication will also continue to affect our MDT meetings in radiosurgery, and as such, there is a unique opportunity in this space for the incorporation of XR technologies to improve our remote case discussions and communication. Furthermore, there is great potential for the amalgamation of machine learning and artificial intelligence with the field of XR in medicine. Machine learning can help deepen our ability to more accurately segment MRI scans and coregister images in AR and MR settings. The horizon for innovation in this field is bright and full of opportunities for more technological innovation.

## Appendix

Multimedia Appendix 1 Demonstration of the key features of the NeuroVis extended reality application system.

## Abbreviations

AR: augmented reality
CT: computed tomography
MDT: multidisciplinary team
MR: mixed reality
MRI: magnetic resonance imaging
MRTK: Mixed Reality Toolkit Library
OAR: organ at risk
PTV: planning treatment volume
SRS: stereotactic radiosurgery
VS: vestibular schwannoma
XR: extended reality
